# Early environmental factors and somatic comorbidity in schizophrenia and nonschizophrenic psychoses: A 50-year follow-up of the Northern Finland Birth Cohort 1966

**DOI:** 10.1192/j.eurpsy.2020.25

**Published:** 2020-02-21

**Authors:** Hanna Korpela, Jouko Miettunen, Nina Rautio, Matti Isohanni, Marjo-Riitta Järvelin, Erika Jääskeläinen, Juha Auvinen, Sirkka Keinänen-Kiukaanniemi, Tanja Nordström, Jussi Seppälä

**Affiliations:** 1 Center for Life Course Health Research, University of Oulu, Oulu, Finland; 2 Cancer Research and Translational Medicine Research Unit, University of Oulu, Oulu, Finland; 3 Medical Research Center Oulu, Oulu University Hospital and University of Oulu, Oulu, Finland; 4 Unit of Primary Health Care, Oulu University Hospital, Oulu, Finland; 5 Department of Epidemiology and Biostatistics, MRC–PHE Center for Environment & Health, School of Public Health, Imperial College London, London, United Kindom; 6 Biocenter Oulu, University of Oulu, Oulu, Finland; 7 Department of Psychiatry, Oulu University Hospital, Oulu, Finland; 8 Infrastructure for Population Studies, Faculty of Medicine, University of Oulu, Oulu, Finland; 9 Department of Psychiatry and Substance Use, South Karelia Social and Health Care District, Lappeenranta, Finland

**Keywords:** cohort study, physical illnesses, population-based, psychosis, schizophrenia, somatic comorbidity

## Abstract

**Background.:**

We studied the cumulative incidence of physical illnesses, and the effect of early environmental factors (EEFs) on somatic comorbidity in schizophrenia, in nonschizophrenic psychosis and among nonpsychotic controls from birth up to the age of 50 years.

**Methods.:**

The sample included 10,933 members of the Northern Finland Birth Cohort 1966, of whom, 227 had schizophrenia and 205 had nonschizophrenic psychosis. Diagnoses concerning physical illnesses were based on nationwide registers followed up to the end of 2016 and classified into 13 illness categories. Maternal education and age, family type at birth and paternal socioeconomic status were studied as EEFs of somatic illnesses.

**Results.:**

When adjusted by gender and education, individuals and especially women with nonschizophrenic psychosis had higher risk of morbidity in almost all somatic illness categories compared to controls, and in some categories, compared to individuals with schizophrenia. The statistically significant adjusted hazard ratios varied from 1.27 to 2.42 in nonschizophrenic psychosis. Regarding EEFs, single-parent family as the family type at birth was a risk factor for a higher somatic score among men with schizophrenia and women with nonschizophrenic psychosis. Maternal age over 35 years was associated with lower somatic score among women with nonschizophrenic psychosis.

**Conclusions.:**

Persons with nonschizophrenic psychoses have higher incidence of somatic diseases compared to people with schizophrenia and nonpsychotic controls, and this should be noted in clinical work. EEFs have mostly weak association with somatic comorbidity in our study.

## Introduction

Individuals with psychoses and especially with schizophrenia have increased rates of physical illnesses compared to the general population [1–3]. Cardiovascular morbidity and mortality, and the risk for type 2 diabetes are approximately two- to three-fold higher in schizophrenia compared to the general population, and people with schizophrenia have about 10–25 years shorter life expectancy compared to the general population [3–7].

Schizophrenia is associated with increased incidence of cardiovascular disease, stroke, and coronary heart disease [4,8]. Still, these patients receive less antihypertensive and lipid-lowering treatments [9]. Additionally, metabolic syndrome, overweight, hyperglycemia, and lipid abnormalities are common in patients with schizophrenia [10].

Higher comorbidity is linked to various factors, for example, the disorder itself and its consequences (e.g., lifestyle), medication use or neglect by the medical profession regarding adequate screening and treatment [10,11]. Also, those with schizophrenia, bipolar disorder, or major depressive disorder are more sedentary and less physically active than nonpsychotic controls [11].

The effect of early environmental factors (EEFs), such as maternal age and education, family type and paternal socioeconomic status (SES) at birth, on somatic comorbidity, or health in general has been studied in nonpsychotic samples [12–15], but not in psychotic samples. Previous studies show that parental SES is associated with offspring health, and low parental education, maternal age, and growing up in a single-parent family are risk factors for poor health outcomes [12–17].

Compared to schizophrenia, somatic illnesses in nonschizophrenic psychosis are not well studied. The aim of this study was to evaluate the prevalence of physical illnesses among individuals with schizophrenia, nonschizophrenic psychosis, and nonpsychotic controls up to the age of 50 years in the prospective Northern Finland Birth Cohort 1966 (NFBC 1966). We also aimed to analyze potential EEFs’ effects on somatic comorbidity in psychoses. Our hypothesis was that somatic comorbidity is higher in schizophrenia than in nonschizophrenic psychosis or among nonpsychotic controls, as studies show that individuals with schizophrenia have lower physical health in general compared to nonpsychotic individuals [18,19]. To the best of our knowledge, there are only a few previous prospective, unselected birth cohort studies analyzing somatic comorbidities in psychoses. Especially there is a lack of studies comparing somatic comorbidity in schizophrenia and in nonschizophrenic psychosis.

## Methods

### Study design and material

This study is based on the population-based, unselected, and prospective NFBC 1966 concerning 12,058 live-born children in 1966 in the provinces of Lapland and Oulu [20]. The study design and data of NFBC 1966 have been described in detail elsewhere [20,21]. The present study population consists of 10,933 individuals being alive at the age of 16 years and living in Finland. They were followed from birth up to the age of 50 years and diagnoses of somatic and psychotic illnesses were coded from age 16 onwards. The NFBC 1966 study design has been approved by The Ethics Committee of the Northern Ostrobothnia Hospital District.

### Identification of psychoses and somatic illnesses

Psychotic and somatic diagnoses were collected from various nationwide registers: The Care Register for Health Care (CRHC) covering all treatment episodes in mental, general, and military hospitals, and in the inpatient wards of local health centers nationwide for the period up to 2016; Register of Specialty Health Care for the period 1998–2016; Register of Primary Health Care Visits in 2011–2013; the registers of the Social Insurance Institution of Finland (including information on reimbursed medicine up to 2005, pensions up to 2000 and sick days up to the end of 1999); and the register of the Finnish Center for Pensions up to 2013 (data on disability pensions) [22].

### Study population and diagnosis of psychoses

Diagnoses were coded according to the International Classification of Diseases Eighth Revision (ICD-8) before 1987, according to ICD-9 1987–1995, and according to ICD-10 since 1996.

Individuals were classified as having schizophrenia (i.e., disorders of the schizophrenia spectrum: ICD-8: 295; ICD-9: 295, 2954, 2957, 297; ICD-10: F20, F22, F25) or nonschizophrenic psychosis (bipolar disorder with psychotic features, major depressive episode with psychotic features, brief psychosis, and other psychosis: ICD-8: 296-299; ICD-9: 298-299, 2961E, 2962E, 2963E, 2964E, 2967; ICD-10: F23-F24, F26, F29, F30.2, F31.2, F31.5, F32.3, F33.3) or no psychosis.

The study population included 432 individuals (237 men and 195 women) with psychosis and 10 501 nonpsychotic controls (5,352 men and 5,149 women), of which 1896 (17.3%) had a nonpsychotic mental, behavioral or neurodevelopmental disorder. In the psychosis population 227 (53%) had schizophrenia (173 had narrow schizophrenia defined as ICD-10 diagnoses F20.0–F20.9, 54 had other schizophrenia spectrum disorder), and 205 (47%) subjects were diagnosed with nonschizophrenic psychosis (72 subjects with a major depressive episode with psychotic features, 24 with bipolar disorder with psychotic features, and 109 with brief or undefined psychosis) (Table 1). The diagnostic groups within nonschizophrenic psychosis were not analyzed separately due to the low number of cases.

**Table 1.** Gender, education and early environmental factors in individuals with schizophrenia, nonschizophrenic psychosis, and no psychosis in the NFBC 1966 cohort

	Schizophrenia (*n* = 227)		Nonschizophrenic psychosis (*n* = 205)		No psychoses (*n* = 10,501)	
	*n*	%	*n*	%	*n*	%
Gender						
Men	131	57.7	106	51.7	5,352	51.0
Women	96	42.3	99	48.3	5,149	49.0
Education						
Basic (≤9 years)	68	30.0	51	24.9	1,564	14.9
Secondary (10–12 years)	138	60.8	127	62.0	6,246	59.5
Tertiary (over 12 years)	21	9.3	27	13.2	2,679	25.5
Mother’s education						
Low (0–4 years)	14	6.2	18	8.8	954	9.1
Intermediate (5–8 years)	125	55.1	123	60.0	5,871	55.9
High (≥9 years)	88	38.8	64	31.2	3,675	35.0
Mother’s age at birth						
<20 years	18	7.9	18	8.8	991	9.4
20–35 years	172	75.8	158	77.1	8,010	76.3
>35 years	37	16.3	29	14.1	1,499	14.3
Paternal socioeconomic status at birth						
High (I–II)	21	9.3	8	3.9	760	7.3
Low (III–IV)	170	74.9	153	74.6	7,685	73.2
Farmers (V)	35	15.4	44	21.5	2,009	19.1
Family type at birth						
Two parent family	213	93.8	193	94.1	10,095	96.3
Single-parent family	14	6.2	12	5.9	390	3.7

### Diagnoses of somatic illnesses

Based on information from CRHC, physical illnesses were classified according to ICD-10 Chapters I–XIV and all the diagnoses at age 16 and onwards were considered by transforming ICD-8 and ICD-9 diagnoses to ICD-10 diagnoses. Of the ICD-10, Chapter V Mental and Behavioral Disorders was excluded. Chapters from XIV onwards were excluded because of their nature, as they include, for example, injuries, poisoning, and external causes of morbidity and mortality. The included 13 somatic illness categories are shown in Table 2, and the diagnostic codes used to identify these categories are presented in Table S1.

**Table 2.** Somatic comorbidity in men and women with schizophrenia, nonschizophrenic psychoses, and nonpsychotic controls

Somatic diseases	Schizophrenia (*n* = 227)					Nonschizophrenic psychoses (*n* = 205)					Nonpsychotic controls (*n* = 10,501)	
	*n*	%	AHR	95% CI	BH *p*-value	*n*	%	AHR	95% CI	BH *p*-value	*n*	%
1. Certain infectious and parasitic diseases	60	26.4	1.35	1.04–1.75	0.07	64	31.2	**1.68**	**1.31–2.16**	**0.0001**	2,013	19.2
2. Neoplasms	54	23.8	1.33	1.01–1.74	0.11	40	19.5	1.01	0.74–1.38	0.96	2,118	20.2
3. Diseases of the blood and blood-forming organs and certain disorders involving the immune mechanism	18	7.9	**2.00**	**1.25–3.22**	**0.02**	19	9.3	**2.29**	**1.45–3.64**	**0.0008**	425	4.0
4. Endocrine, nutritional, and metabolic diseases	51	22.5	**1.81**	**1.36–2.39**	**0.0005**	58	28.3	**2.42**	**1.86–3.15**	**<0.0001**	1,330	12.7
5. Diseases of the nervous system	55	24.2	1.08	0.83–1.41	0.62	70	34.1	**1.61**	**1.27–2.05**	**0.0002**	2,345	22.3
6. Diseases of the eye and adnexa	42	18.5	1.19	0.88–1.62	0.35	34	16.6	1.04	0.74–1.46	0.91	1,739	16.6
7. Diseases of the ear and mastoid process	30	13.2	1.23	0.86–1.77	0.35	30	14.6	1.40	0.97–2.01	0.09	1,149	10.9
8. Diseases of the circulatory system	67	29.5	1.23	0.97–1.57	0.20	85	41.5	**1.53**	**1.23–1.90**	**0.0003**	2,804	26.7
9. Diseases of the respiratory system	114	50.2	1.04	0.86–1.25	0.67	120	58.5	**1.27**	**1.06–1.52**	**0.01**	5,014	47.7
10. Diseases of the digestive system	143	63.0	1.14	0.96–1.34	0.24	137	66.8	**1.31**	**1.10–1.55**	**0.003**	6,233	59.4
11. Diseases of the skin and subcutaneous tissue	51	22.5	1.16	0.88–1.53	0.35	65	31.7	**1.72**	**1.34–2.20**	**0.0001**	2,014	19.2
12. Diseases of the musculoskeletal system and connective tissue	85	37.4	**0.68**	**0.54–0.84**	**0.002**	113	55.1	1.21	1.00–1.45	0.06	5,117	48.7
13. Diseases of the genitourinary system	74	32.6	1.14	0.90–1.43	0.35	89	43.4	**1.59**	**1.29–1.96**	**0.0001**	3,193	30.4

Bold values are statistically significant, *p*-value < 0.05. Abbreviations: AHR, adjusted hazard ratio; BH *p*-value, Benjamini–Hochberg corrected *p*-value; 95% CI, 95% confidence interval.

### Somatic score

“Somatic score” for each cohort member was calculated based on somatic illness diagnoses. All the diagnoses within one somatic illness category gave a maximum of 1 point and given points from different illness categories were summed. A person may have numerous diagnoses within any given illness category, but the score for the category remains 1. The score for somatic diseases ranged from 0 to 13.

### Covariates and early environmental factors for somatic illnesses

Gender and education were analyzed as confounders for somatic illnesses. These were selected based on earlier studies [1,18] and our data (see section “Statistical analyses”). Education data were obtained from the Finnish Education Register 1997, Statistics Finland and classified as basic: less than 9 years; secondary: 9–12 years; and tertiary: more than 12 years.

The selected EEFs were maternal education and age, family type at birth and paternal SES. These have been associated with increased risk of psychoses [23–28] and increased risk of somatic illnesses in nonpsychotic samples [12–17]. Data were gathered from the Population Register Center and from questionnaires during the mothers’ visits to antenatal clinics at 24–28 weeks gestation. Data for maternal education was obtained from questionnaires and classified as low: 0–4 years, intermediate: 5–8 years and high education: more than 8 years [29]. Data for maternal age was obtained from the Population Register Center and classified as under 20 years, 20–35 years, and over 35 years [29]. Family type at birth was obtained from the questionnaire and based on the marital status of the mother during pregnancy (married, divorced, widowed, or never married). Family type was classified as two-parent families and single-parent families [30]. SES at birth was obtained from the questionnaire and based on the father’s occupation. SES was classified as high (classes I and II), low (classes III and IV) and farmers (class V) [30].

### Statistical analyses

The background variables were compared between individuals with schizophrenia, nonschizophrenic psychosis, and controls by using cross-tabulation and the *χ*^2^-exact test. Cox regression analysis was used to examine the risk of physical illness in schizophrenia and nonschizophrenic psychosis compared to the nonpsychotic controls.

Cox regression analyses were done unadjusted and adjusted for gender and education, and adjusted for EEFs. Additionally, analyses were done in strata by gender as women and men may have gender differences in health and in symptom reporting [31,32].

We present the results as adjusted hazard ratios for gender and education (AHRs) with 95% confidence intervals (95% Cls). Unadjusted hazard ratios (HR) are presented if they differ significantly from AHRs. Cox regression analyses adjusted for EEFs were in line with presented AHRs. Benjamini–Hochberg procedure was used to correct for multiple comparisons and Benjamini–Hochberg corrected *p*-values (BH *p*-value) are presented.

Differences in the somatic score between individuals with schizophrenia, nonschizophrenic psychosis, and controls were examined by using Mann–Whitney’s *U* test and Kruskal–Wallis *H* test. Effect of gender and EEFs on somatic score was tested by a one-way analysis of variance, or by the Brown–Forsythe test when group variances within an EEF were not statistically equal. Effect sizes for EEFs were calculated using Hedges’ *g* and results were interpreted with the following cut-offs: small effect size = 0.2, medium effect size = 0.5, and large effect size = 0.8. Control groups for effect size were high maternal education, maternal age of 20–35 years, high SES and two-parent family. Analyses were performed using IBM SPSS Statistics 24.0.

## Results

### Characteristics of the sample

The distribution between genders was rather equal, though there were more men than women with schizophrenia (57.7 vs. 42.3%). Most individuals have completed secondary education, though tertiary education was more common among nonpsychotic controls (25.5%) than among people with schizophrenia (9.3%) and nonschizophrenic psychosis (13.2%) (Table 1).

### Risk of somatic illnesses in schizophrenia

Diseases of the blood and blood-forming organs (7.9% in schizophrenia vs. 4.0% in nonpsychotic controls; AHR: 2.00; 95% CI: 1.25–3.22) and endocrine, nutritional, and metabolic diseases (22.5 vs. 12.7%; AHR: 1.81; 95% CI: 1.36–2.39) were more common among individuals with schizophrenia compared to controls (Table 2). Diseases of the musculoskeletal system and connective tissue were less common in schizophrenia compared to controls (37.4 vs. 48.7%; AHR: 0.68; 95% CI: 0.54–0.84). Adjusted results were in line with unadjusted results except for certain infectious and parasitic diseases, in which prevalence was higher in schizophrenia (26.4 vs. 19.2%; HR: 1.46; 95% CI: 1.13–1.88) in unadjusted analyses.

When analyzed in strata by gender, men with schizophrenia had higher prevalence of diseases of the blood and blood-forming organs, endocrine, nutritional, and metabolic diseases and diseases of the genitourinary system. Diseases of the musculoskeletal system and connective tissue were lower compared to the controls (Tables 3 and 4). The unadjusted results were in line with the adjusted results except for diseases of the musculoskeletal system and connective tissue which did not have a statistically significant difference in men with or without schizophrenia in unadjusted analyses.

**Table 3.** Somatic comorbidity in men with schizophrenia, nonschizophrenic psychoses, and nonpsychotic controls

Somatic diseases	Schizophrenia (*n* = 131)					Nonschizophrenic psychoses (*n* = 106)					Nonpsychotic controls (*n* = 5,352)	
	*n*	%	AHR	95% CI	BH *p*-value	*n*	%	AHR	95% CI	BH *p*-value	*n*	%
1. Certain infectious and parasitic diseases	38	29.0	1.35	0.98–1.87	0.14	31	29.2	1.38	0.96–1.97	0.13	1,179	22.0
2. Neoplasms	20	15.3	1.34	0.86–2.09	0.32	11	10.4	0.90	0.50–1.64	0.74	658	12.3
3. Diseases of the blood and blood-forming organs and certain disorders involving the immune mechanism	10	7.6	**2.88**	**1.51–5.48**	**0.01**	8	7.5	**2.89**	**1.42–5.90**	**0.01**	145	2.7
4. Endocrine, nutritional, and metabolic diseases	28	21.4	**1.97**	**1.35–2.89**	**0.01**	21	19.8	**1.88**	**1.22–2.91**	**0.01**	604	11.3
5. Diseases of the nervous system	30	22.9	1.19	0.83–1.71	0.45	32	30.2	**1.64**	**1.15–2.33**	**0.01**	1,054	19.7
6. Diseases of the eye and adnexa	15	12.1	1.23	0.80–1.88	0.45	13	12.3	0.90	0.52–1.56	0.74	790	14.8
7. Diseases of the ear and mastoid process	19	15.3	1.10	0.66–1.85	0.77	13	12.3	1.22	0.70–2.12	0.62	572	10.7
8. Diseases of the circulatory system	31	25.0	1.38	0.99–1.92	0.14	44	41.5	**1.89**	**1.40–2.56**	**0.0004**	1,352	25.3
9. Diseases of the respiratory system	75	60.5	1.01	0.80–1.28	0.94	63	59.4	1.05	0.82–1.35	0.74	2,938	54.9
10. Diseases of the digestive system	64	51.6	1.17	0.94–1.47	0.29	67	63.3	**1.37**	**1.08–1.75**	**0.02**	3,141	58.7
11. Diseases of the skin and subcutaneous tissue	22	17.7	1.12	0.76–1.64	0.68	34	32.1	**1.91**	**1.36–2.69**	**0.001**	979	18.3
12. Diseases of the musculoskeletal system and connective tissue	36	29.0	**0.69**	**0.52–0.92**	**0.04**	56	52.8	1.23	0.94–1.60	0.18	2,520	47.1
13. Diseases of the genitourinary system	25	20.2	**1.87**	**1.28–2.73**	**0.01**	23	21.7	1.90	1.25–2.88	0.01	669	12.5

Bold values are statistically significant, *p*-value < 0.05. Abbreviations: AHR, adjusted hazard ratio; BH *p*-value, Benjamini–Hochberg corrected *p*-value; 95% CI, 95% confidence interval.

**Table 4.** Somatic comorbidity in women with schizophrenia, nonschizophrenic psychoses, and nonpsychotic controls

Somatic diseases	Schizophrenia (*n* = 96)					Nonschizophrenic psychoses (*n* = 99)					Nonpsychotic controls (*n* = 5,149)	
	*n*	%	AHR	95% CI	BH *p*-value	*n*	%	AHR	95% CI	BH *p*-value	*n*	%
1. Certain infectious and parasitic diseases	22	22.9	1.32	0.86–2.01	0.58	33	33.3	**2.12**	**1.49–3.00**	**0.0002**	834	16.2
2. Neoplasms	34	35.4	1.32	0.94–1.85	0.49	29	29.3	1.06	0.73–1.52	0.77	1460	28.4
3. Diseases of the blood and blood-forming organs and certain disorders involving the immune mechanism	8	8.3	1.45	0.71–2.93	0.66	11	11.1	**1.99**	**1.09–3.64**	**0.05**	280	5.4
4. Endocrine, nutritional, and metabolic diseases	23	24.0	1.64	1.08–2.49	0.13	37	37.4	**2.89**	**2.07–4.02**	**<0.0001**	726	14.1
5. Diseases of the nervous system	25	26.0	0.97	0.65–1.44	0.88	38	38.4	**1.59**	**1.15–2.20**	**0.01**	1291	25.1
6. Diseases of the eye and adnexa	20	20.8	1.14	0.73–1.78	0.68	21	21.2	1.13	0.74–1.75	0.62	949	18.4
7. Diseases of the ear and mastoid process	15	15.6	1.38	0.82–2.30	0.58	17	17.2	1.56	0.96–2.53	0.11	577	11.2
8. Diseases of the circulatory system	29	30.2	1.08	0.74–1.56	0.75	41	41.4	1.27	0.93–1.73	0.17	1,452	28.2
9. Diseases of the respiratory system	44	45.8	1.10	0.82–1.49	0.68	57	57.6	**1.68**	**1.29–2.18**	**0.0005**	2,076	40.3
10. Diseases of the digestive system	63	65.6	1.09	0.85–1.40	0.68	70	70.7	1.24	0.98–1.57	0.11	3,092	60.1
11. Diseases of the skin and subcutaneous tissue	24	25.0	1.21	0.81–1.82	0.66	31	31.3	**1.54**	**1.08–2.20**	**0.04**	1,035	20.1
12. Diseases of the musculoskeletal system and connective tissue	38	39.6	0.65	0.47–0.90	0.11	57	57.6	1.18	0.91–1.53	0.26	2,597	50.4
13. Diseases of the genitourinary system	46	47.9	0.92	0.69–1.23	0.68	66	66.7	**1.50**	**1.18–1.92**	**0.004**	2,524	49.0

Bold values are statistically significant, *p*-value < 0.05. Abbreviations: AHR, adjusted hazard ratio; BH *p*-value, Benjamini–Hochberg corrected *p*-value; 95% CI, 95% confidence interval.

### Risk of somatic illnesses in nonschizophrenic psychoses

Diseases in multiple somatic illness categories were more common among individuals with nonschizophrenic psychosis compared to nonpsychotic controls (Table 2)**.** Diseases of the blood and blood-forming organs (9.3 vs. 4.0%; AHR: 2.29; 95% CI: 1.45–3.64) and endocrine, nutritional, and metabolic diseases (28.31 vs. 12.7%; AHR: 2.42; 95% CI: 1.86–3.15) were significantly more prevalent in nonschizophrenic psychosis than in controls (Tables 3 and 4). The unadjusted results were in line with the adjusted results except for the diseases of the musculoskeletal system and connective tissue, which had higher prevalence among individuals with nonschizophrenic psychosis when analyses were done unadjusted (55.1 vs. 48.7%; HR: 1.26; 95% CI: 1.04–1.52).

When analyzed in strata by gender, men with nonschizophrenic psychosis had significantly higher prevalence of diseases of the blood and blood-forming organs (7.5 vs. 2.7%; AHR: 2.89; 95% CI: 1.42–5.90) than male controls. Women with nonschizophrenic psychosis had significantly higher prevalence of endocrine, nutritional, and metabolic diseases (37.4 vs. 14.1%; AHR: 2.89; 95% CI: 2.07–4.02) compared to female controls. Disease prevalence was statistically significantly higher in various disease categories as seen in Tables 3 and 4. Unadjusted results were in line with adjusted results except for diseases of the digestive system among women with nonschizophrenic psychosis where prevalence was higher compared to female controls when analyzed unadjusted (70.7 vs. 60.1%; HR: 1.31; 95% CI: 1.03–1.66).

### The risk of somatic illness in schizophrenia compared to nonschizophrenic psychoses

Compared to nonschizophrenic psychosis, individuals with schizophrenia had less diseases of the nervous system (24.2 vs. 34.1%; AHR: 0.66; 95% CI: 0.46–0.94), diseases of the skin and subcutaneous tissue (22.5 vs. 31.7%; AHR: 0.66; 95% CI: 0.46–0.96), diseases of the musculoskeletal system and connective tissue (37.4 vs. 55.1%; AHR: 0.56; 95% CI: 0.42–0.74) and diseases of the genitourinary system (32.6 vs. 43.4%; AHR: 0.69; 95% CI: 0.50–0.93).

### Somatic score

**Figure 1.**
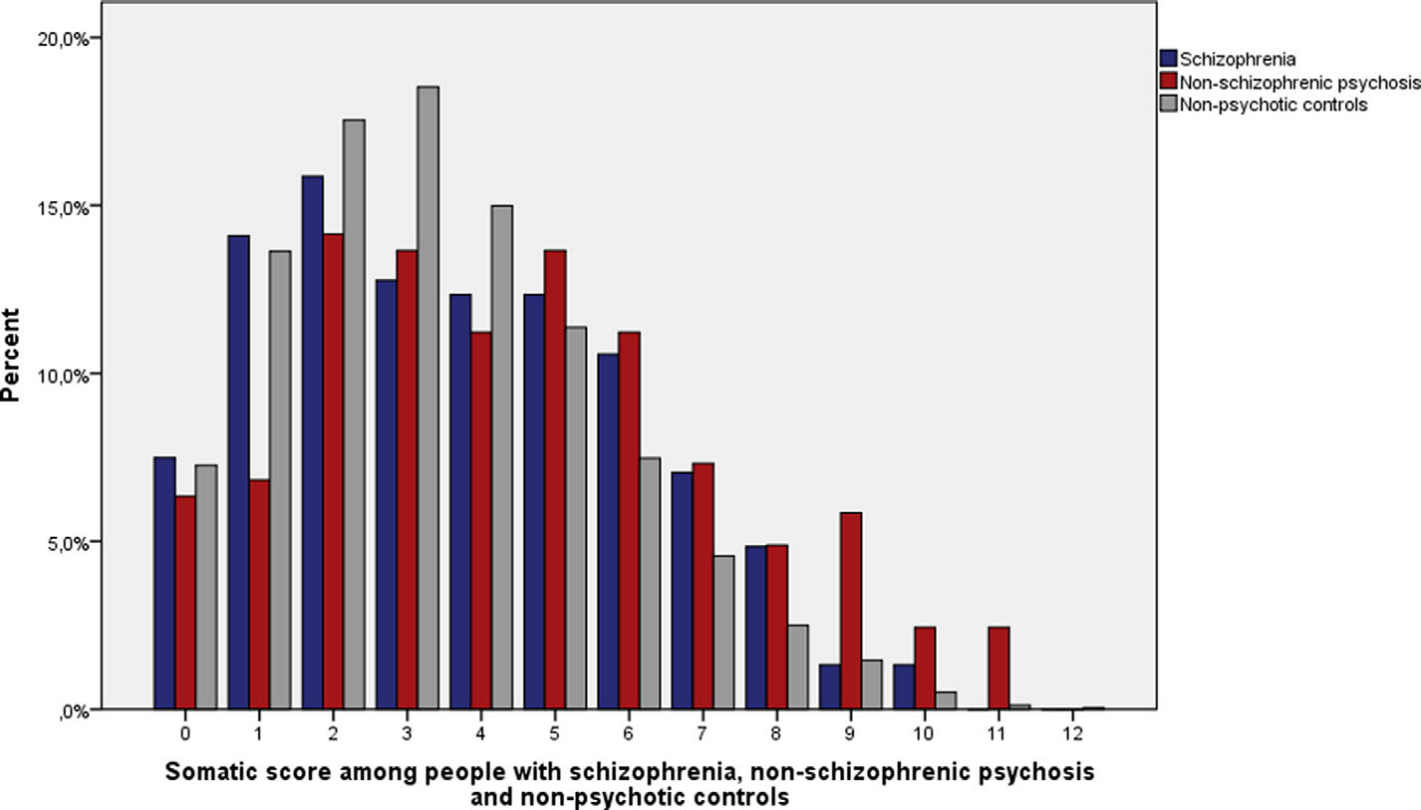
Distribution of somatic score among people with schizophrenia (*n* = 227), nonschizophrenic psychosis (*n* = 205), and nonpsychotic controls (*n* = 10,501).

When both sexes were examined together, the median number of somatic diseases from different somatic illness categories was three among people with schizophrenia, four among people with nonschizophrenic psychosis and three among nonpsychotic controls (Figure 1)**.** The difference in somatic score in people with nonschizophrenic psychosis compared to both schizophrenia (*U* = 26,917; *p* = 0.005) and nonpsychotic controls (*U* = 825,117; *p* < 0.001) was statistically significant. The difference of the distribution of somatic diseases between people with schizophrenia and nonpsychotic controls (*U* = 1,104,804; *p* = 0.06) was nonsignificant.

The median somatic score was 3 among men with schizophrenia and controls, and 4 among men with nonschizophrenic psychosis. The median somatic score was 4 among women with schizophrenia, 5 among women with nonschizophrenic psychosis, and 3 among control females. Women with nonschizophrenic psychosis had statistically significantly higher median somatic scores compared to men with nonschizophrenic psychosis (*U* = 6,520; *p* = 0.003).

### Early environmental factors of somatic illness

Table S2 describes the median and mean somatic scores among different EEFs according to sex and psychotic illness group. Among women with schizophrenia and men with nonschizophrenic psychosis, none of the EEFs predicted the somatic score.

Single-parent family as the family type at birth was a risk factor for higher somatic score among men with schizophrenia (median somatic score = 5 in single-parent family vs. 3 in two-parent family; *p* = 0.01; Hedges’ *g* = 0.86) and among women with nonschizophrenic psychosis (median somatic score = 7 vs. 5; *p* = 0.03; Hedges’ *g* = 0.87).

Among women with nonschizophrenic psychosis, maternal age higher than 35 years was associated with lower somatic score (Median somatic score = 3 vs. 5; *p* = 0.03; Hedges’ *g* = 0.76) compared to the reference group of mothers (age 20–35).

Among controls, high maternal education and high SES statistically significant predictors of lower somatic score among both men and women. Among control women, young maternal age was also a statistically significant predictor of higher somatic score. Effect sizes among statistically significant predictors varied from 0.002 to 0.22, so, despite the statistical significance, EEFs were weak predictors for somatic illness among controls in our study population.

## Discussion

### Main findings

The main finding in our study was that people with nonschizophrenic psychosis had increased risk of several somatic illnesses compared to schizophrenia or nonpsychotic controls. People with nonschizophrenic psychosis had more diseases of the skin, nervous, genitourinary, and musculoskeletal system than people with schizophrenia. Especially women with nonschizophrenic psychosis had a higher risk of somatic comorbidities. EEFs had a very small effect among nonpsychotic individuals. Among men with schizophrenia and women with nonschizophrenic psychosis, single-parent family as the family type at birth was a significant risk factor for higher somatic comorbidity. Among women with nonschizophrenic psychosis, maternal age higher than 35 years at birth seemed to be a protective factor against somatic illnesses.

### Comparison with earlier studies

Some previous studies show that somatic comorbidity is common among patients with depressive disorder and that the prevalence of somatic illnesses is more common in patients with bipolar disorder than among patients with schizophrenia [33–35]. People with schizoaffective disorder have a higher risk of metabolic syndrome than people with schizophrenia or other nonaffective psychoses [36]. It may be that especially depressive symptoms increase the risk of metabolic syndrome and somatic illnesses via unhealthy lifestyle, medication, and co-occurring biological mechanisms (e.g., hypercortisolism) [35,36]. These may be some of the reasons behind our findings of higher risk of somatic comorbidity among nonschizophrenic psychoses, as suggested by some previous literature [11]. In addition, persons with nonschizophrenic psychoses, especially those with affective psychosis, may have more personality problems or impulsiveness that associate to, for example, smoking and alcohol use, and somatic problems relating to those [37]. Persons with nonschizophrenic psychoses may also seek medical help more often compared to persons with schizophrenia. Thus, there may be some undiagnosed somatic comorbidities in the schizophrenia group [38].

Women with nonschizophrenic psychosis had a higher risk of somatic comorbidities in our sample. Some studies have found evidence that women experience multiple comorbidities in schizophrenia and psychosis [6,33], and they are more ready to report illness and to seek help than men [39,40]. Women have more morbidity burden at all ages, and they experience the negative side-effects of antipsychotics (e.g., weight gain, diabetes, and cardiovascular risks) more than men [31,32,41,42].

Diseases of the blood and blood-forming organs and disorders of the immune mechanism were more prevalent in subjects with psychosis compared to nonpsychotic controls. One explanation for this could be altered development of the immune system, which has been linked to development of psychosis and other psychiatric disorders [43,44].

Our study shows an association between endocrine, nutritional, and metabolic diseases and psychoses, the finding being consistent with previous studies that have shown associations especially between metabolic syndrome, diabetes, and thyroid dysfunction and psychoses [45-47].

Several studies have suggested that schizophrenia is associated with increased incidence of cardiovascular disease and coronary heart disease [9,48], but also contrary results have been found [6,49]. In our study, there was higher prevalence of diseases of the circulatory system only among men with nonschizophrenic psychosis. This could be because of the relatively young age of our study population, as the risk for cardiovascular diseases increases with age [50].

In our study, diseases of the musculoskeletal system and connective tissue were less common in men with schizophrenia than in controls. Studies have shown that there is a reduced risk of musculoskeletal diseases in schizophrenia and in schizoaffective patients [33,51]. This may be due to pain insensitivity to chronic pain, as people with schizophrenia have decreased or altered pain perception [52] and the lack of pain might leave physical diseases undiagnosed.

### Early environmental factors and somatic illnesses

The only significant predictors of somatic illnesses among individuals with psychosis were family type at birth and maternal age.

It has been shown that children living in single-parent families have poorer health than children living with two biological parents [14,53]. Our study results support this finding partially as single-parent family was a risk factor of somatic illnesses among men with nonschizophrenic psychosis and women with schizophrenia.

Studies show that offspring born to mothers younger than 25 years or older than 35 years have worse outcomes with respect to, for example, self-rated health and the number of diagnosed conditions as adults than those born to mothers aged 25–34 [12]. Our results are contrary, as higher maternal age was associated with lower somatic comorbidity among women with nonschizophrenic psychosis.

### Limitations

Regarding somatic illnesses, our sample may include mainly the patients who have primarily been in hospital care because the outpatient data from primary health care is available only from 2011 onwards and from specialized outpatient care from 1998 onwards. Thus, we may have missed some of the less severe somatic diagnoses and some psychosis cases treated solely as outpatients before 1998. On the other hand, in earlier days, most psychosis cases were hospitalized [22], and Finnish National Registries have been found to be reliable sources for case detection in severe psychotic disorders [54,55]. The limitations also include the lack of clinical details and generalizability of the results to populations other than Northern Europe. The study was not designed for the purpose of looking back at data.

Neither psychiatric nor somatic medications were included in our data, and as medication might have some adverse effects on one’s health, it causes study bias. Due to follow-up up to middle age, the generalizability of the results to older age groups is limited. Limitations include the relatively young age of the participants at the end of the follow-up and the low number of cases in some of the somatic illness groups.

Somatic score is a rough measure and may cause bias as it gives the same somatic score regardless of how many diagnoses one has within one somatic illness category. It neither describes the severity of illnesses nor the severity of one’s condition. Somatic score has not been used before.

## Conclusion

Our results suggest that people with nonschizophrenic psychosis show a greater occurrence of somatic diseases compared to nonpsychotic controls, and this should be noted by medical professionals. Further studies are warranted to investigate somatic comorbidities and their causes in nonschizophrenic psychosis and longitudinal studies on risk factors of somatic comorbidities in schizophrenia and nonschizophrenic psychosis during lifespans are needed.

## Supplementary material

For supplementary material accompanying this paper visit http://dx.doi.org/10.1192/j.eurpsy.2020.25. click here to view supplementary material

The data that support the findings of this study are available from the Northern Finland Cohorts (www.oulu.fi/nfbc) for research purposes with a study plan.
