# Supplementary material for: Early environmental factors and somatic comorbidity in schizophrenia and nonschizophrenic psychoses: A 50-year follow-up of the Northern Finland Birth Cohort 1966
Source: Eur Psychiatry. 2020 Feb 21;63(1):e24. doi: 10.1192/j.eurpsy.2020.25 (PMC7315879; doi:10.1192/j.eurpsy.2020.25)
Supplement: Supplementary file 1 [file S0924933820000255sup.zip › S0924933820000255sup002.docx]

Online supplement table 2. Early environmental factors as predictors of somatic illnesses measured as score of somatic diseases

|  | Schizophrenia (n=227) | | | | | Non-schizophrenic psychoses (n=205) | | | | | Non-psychotic controls (n=10 501) | | | | |
| --- | --- | --- | --- | --- | --- | --- | --- | --- | --- | --- | --- | --- | --- | --- | --- |
|  | n (%) | Md | M | p-value | effect size | n (%) | Md | M | p-value | effect size | n (%) | Md | M | p-value | effect size |
|  |  |  |  |  |  |  |  |  |  |  |  |  |  |  |  |
| **Men** | (n = 131) |  |  |  |  | (n = 106) |  |  |  |  | (n = 5352) |  |  |  |  |
|  |  |  |  |  |  |  |  |  |  |  |  |  |  |  |  |
| *Maternal education* |  |  |  |  |  |  |  |  |  |  |  |  |  |  |  |
|  |  |  |  |  |  |  |  |  |  |  |  |  |  |  |  |
| Low (0-4 years) | 6 (4.6) | 3 | 3.50 |  | 0.15 | 10 (9.4) | 4 | 3.60 |  | 0.13 | 465 (8.7) | 3 | 2.98 |  | **0.002** |
|  |  |  |  |  |  |  |  |  |  |  |  |  |  |  |  |
| Intermediate (5-8 years) | 82 (62.6) | 3 | 3.23 | 0.43 | 0.24 | 66 (62.3) | 4 | 4.26 | .21 | 0.39 | 3039 (56.8) | 3 | 3.19 | **.001** | **0.10** |
|  |  |  |  |  |  |  |  |  |  |  |  |  |  |  |  |
| High (> 9 years) | 43 (32.8) | 4 | 3.84 |  | - | 30 (28.3) | 3 | 3.30 |  | - | 1848 (34.5) | 3 | 2.99 |  | - |
|  |  |  |  |  |  |  |  |  |  |  |  |  |  |  |  |
| *Maternal age* |  |  |  |  |  |  |  |  |  |  |  |  |  |  |  |
|  |  |  |  |  |  |  |  |  |  |  |  |  |  |  |  |
| < 20 years | 11 (8.4) | 4 | 4.82 |  | 0.59 | 12 (11.3) | 5 | 5.08 |  | 0.50 | 509 (9.5) | 3 | 3.05 |  | 0.03 |
|  |  |  |  |  |  |  |  |  |  |  |  |  |  |  |  |
| 20 - 35 years | 97 (74.0) | 3 | 3.39 | .17* | - | 79 (74.5) | 3 | 3.82 | .22 | - | 4078 (76.2) | 3 | 3.11 | .83 | - |
|  |  |  |  |  |  |  |  |  |  |  |  |  |  |  |  |
| > 35 years | 23 (17.6) | 3 | 3.09 |  | 0.13 | 15 (14.2) | 2 | 3.53 |  | 0.12 | 765 (14.3) | 3 | 3.10 |  | 0.006 |
|  |  |  |  |  |  |  |  |  |  |  |  |  |  |  |  |
| *Paternal socioeconomic status at birth* |  |  |  |  |  |  |  |  |  |  |  |  |  |  |  |
| High (I-II) | 13 (9.9) | 3 | 3.54 |  | - | 5 (4.7) | 2 | 2.20 |  | - | 396 (7.4) | 3 | 2.82 |  | - |
|  |  |  |  |  |  |  |  |  |  |  |  |  |  |  |  |
| Low (III-IV) | 99 (75.6) | 3 | 3.58 | .41 | 0.02 | 76 (71.7) | 4 | 4.28 | .05 | 0.38 | 3945 (73.7) | 3 | 3.11 | **.01** | **0.14** |
|  |  |  |  |  |  |  |  |  |  |  |  |  |  |  |  |
| Farmers (V) | 19 (14.5) | 2 | 2.79 |  | 0.35 | 25 (23.6) | 3 | 3.20 |  | 0.47 | 990 (18.5) | 3 | 3.15 |  | **0.17** |
|  |  |  |  |  |  |  |  |  |  |  |  |  |  |  |  |
|  |  |  |  |  |  |  |  |  |  |  |  |  |  |  |  |
| *Family type at birth* |  |  |  |  |  |  |  |  |  |  |  |  |  |  |  |
|  |  |  |  |  |  |  |  |  |  |  |  |  |  |  |  |
|  |  |  |  |  |  |  |  |  |  |  |  |  |  |  |  |
| Two parent family | 121 (92.4) | 3 | 3.31 | **.01** | **-** | 101 (95.3) | 3 | 3.88 | .43 | - | 5154 (96.3) | 3 | 3.09 | .05 | - |
|  |  |  |  |  |  |  |  |  |  |  |  |  |  |  |  |
| Single parent family | 10 (7.6) | 5 | 5.30 |  | **0.86** | 5 (4.7) | 5 | 4.80 |  | 0.36 | 194 (3.7) | 3 | 3.38 |  | 0.14 |
|  |  |  |  |  |  |  |  |  |  |  |  |  |  |  |  |
| **Women** | n = 96 |  |  |  |  | n = 99 |  |  |  |  | n = 5148 |  |  |  |  |
|  |  |  |  |  |  |  |  |  |  |  |  |  |  |  |  |
| *Maternal education* |  |  |  |  |  |  |  |  |  |  |  |  |  |  |  |
|  |  |  |  |  |  |  |  |  |  |  |  |  |  |  |  |
| Low (0-4 years) | 8 (8.3) | 4 | 2.75 |  | 0.62 | 8 (8.1) | 5 | 5.25 |  | 0.02 | 489 (9.5) | 4 | 3.88 |  | **0.22** |
|  |  |  |  |  |  |  |  |  |  |  |  |  |  |  |  |
| Intermediate (5-8 years) | 43 (44.8) | 4 | 4.05 | .26 | 0.11 | 57 (57.6) | 5 | 5.00 | .87 | 0.11 | 2832 (55.0) | 4 | 3.82 | **<.001** | **0.18** |
|  |  |  |  |  |  |  |  |  |  |  |  |  |  |  |  |
| High (> 9 years) | 45 (46.9) | 5 | 4.33 |  | - | 34 (34.3) | 5 | 5.32 |  | - | 1827 (35.5) | 3 | 3.39 |  | - |
|  |  |  |  |  |  |  |  |  |  |  |  |  |  |  |  |
| *Maternal age* |  |  |  |  |  |  |  |  |  |  |  |  |  |  |  |
|  |  |  |  |  |  |  |  |  |  |  |  |  |  |  |  |
| < 20 years | 7 (7.3) | 5 | 4.86 |  | 0.27 | 6 (6.1) | 5 | 4.33 |  | **0.42** | 482 (9.4) | 4 | 3.90 |  | **0.11** |
|  |  |  |  |  |  |  |  |  |  |  |  |  |  |  |  |
| 20-35 years | 75 (78.1) | 4 | 4.17 | .26 | - | 79 (79.8) | 5 | 5.51 | **.03** | **-** | 3932 (76.4) | 3 | 3.63 | **.048** | - |
|  |  |  |  |  |  |  |  |  |  |  |  |  |  |  |  |
| > 35 years | 14 (14.6) | 3 | 3.14 |  | 0.41 | 14 (14.1) | 3 | 3.36 |  | **0.76** | 734 (14.2) | 3 | 3.72 |  | **0.04** |
|  |  |  |  |  |  |  |  |  |  |  |  |  |  |  |  |
| *Paternal socioeconomic status at birth* |  |  |  |  |  |  |  |  |  |  |  |  |  |  |  |
| High (I-II) | 8 (8.3) | 5 | 4.75 |  | - | 3 (3.0) | 5 | 3.67 |  | - | 364 (7.1) | 3 | 3.37 |  | - |
|  |  |  |  |  |  |  |  |  |  |  |  |  |  |  |  |
| Low (III-IV) | 71 (74.0) | 4 | 4.10 | .50 | 0.25 | 77 (77.8) | 5 | 5.42 | .18 | 0.61 | 3740 (72.6) | 3 | 3.66 | **.03** | **0.14** |
|  |  |  |  |  |  |  |  |  |  |  |  |  |  |  |  |
| Farmers (V) | 16 (16.7) | 4 | 3.50 |  | 0.49 | 19 (19.2) | 4 | 4.21 |  | 0.18 | 1019 (19.9) | 3 | 3.67 |  | **0.13** |
|  |  |  |  |  |  |  |  |  |  |  |  |  |  |  |  |
| *Family type at birth* |  |  |  |  |  |  |  |  |  |  |  |  |  |  |  |
|  |  |  |  |  |  |  |  |  |  |  |  |  |  |  |  |
| Two parent family | 92 (95.8) | 3 | 3.99 | .12 | - | 92 (92.9) | 5 | 4.96 | **.03** | **-** | 4941 (96.0) | 3 | 3.66 | .27 | - |
|  |  |  |  |  |  |  |  |  |  |  |  |  |  |  |  |
| Single parent family | 4 (4.2) | 6 | 6.0 |  | 0.81 | 7 (7.1) | 7 | 7.43 |  | **0.87** | 196 (3.8) | 4 | 3.85 |  | 0.08 |

Md = Median somatic score, M = Mean somatic score, * = p-value has been obtained using Brown-Forsythe test, effect size has been calculated using Hedges’ g
