# Supplementary material for: Early environmental factors and somatic comorbidity in schizophrenia and nonschizophrenic psychoses: A 50-year follow-up of the Northern Finland Birth Cohort 1966
Source: Eur Psychiatry. 2020 Feb 21;63(1):e24. doi: 10.1192/j.eurpsy.2020.25 (PMC7315879; doi:10.1192/j.eurpsy.2020.25)
Supplement: Supplementary file 1 [file S0924933820000255sup.zip › S0924933820000255sup001.docx]

Online supplementary table 1. Diagnostic categories and diagnostic codes according to ICD-8, -9 and -10.

| Somatic disorders | ICD-8 (1968-86) | ICD-9 (1987-95) | ICD-10 (1996-) |
| --- | --- | --- | --- |
| 1. Certain infectious and parasitic diseases | 000-136 | 001-139 | A00-B99 |
| 1. Neoplasms | 140-239 | 140-239 | C00-D48 |
| 1. Diseases of the blood and blood forming organs and certain disorders involving the immune mechanism | 280-289 | 280-289 | D50-D89 |
| 1. Endocrine, nutritional and metabolic diseases | 240-279 | 240-279 | E00-E90 |
| a) diabetes mellitus | 250 | 250 | E10-E14 |
| 1. Diseases of the nervous system | 320-358 | 320-359 | G00-G99 |
| a) epilepsy | 345 | 345 | G40-G41 |
| 1. Diseases of the eye and adnexa | 360-379 | 360-379 | H00-H59 |
| 1. Diseases of the ear and mastoid process | 380-389 | 380-389 | H60-H95 |
| 1. Diseases of the circulatory system | 390-458 | 390-459 | I00-I99 |
| 1. Diseases of the respiratory system | 460-519 | 460-519 | J00-J99 |
| 1. Diseases of the digestive system | 520-577 | 520-579 | K00-K93 |
| 1. Diseases of the skin and subcutaneous tissue | 680-709 | 680-709 | L00-L99 |
| 1. Diseases of the musculoskeletal system and connective tissue | 710-738 | 710-739 | M00-M99 |
| 1. Diseases of the genitourinary system | 580-629 | 580-629 | N00-N99 |
